# miR-182-5p affects human bladder cancer cell proliferation, migration and invasion through regulating Cofilin 1

**DOI:** 10.1186/s12935-019-0758-5

**Published:** 2019-02-28

**Authors:** Fei Wang, Dinglan Wu, Zhanping Xu, Jianxiang Chen, Jiye Zhang, Xiaojuan Li, Shiliang Chen, Fengrong He, Jianbing Xu, Liangju Su, Defan Luo, Shufang Zhang, Weifu Wang

**Affiliations:** 1 Department of Urology, Hainan General Hospital, Haikou, China; 2 Shenzhen Key Laboratory of Viral Oncology, The Clinical Innovation & Research Centre, Shenzhen Hospital, Southern Medical University, Shenzhen, Guangdong Province China; 3 Department of Urology, Foshan Hospital of TCM, Foshan, Guangdong Province China; 4 Department of Urology, Affiliated Hospital of Xiangnan College, Chenzhou, China; 5 Central Laboratory, Hainan General Hospital, Haikou, China; 6 Department of Pathology, Hainan General Hospital, Haikou, China; 7 Central Laboratory, Affiliated Haikou Hospital Xiangya School of Medicine Central South University (HaiKou Municipal People Hospital), Haikou, Hainan China

**Keywords:** miR-182-5p, Bladder cancer, Cofilin 1, Proliferation, Migration, Invasion

## Abstract

**Background:**

Human bladder cancer is one of the common malignant tumors, and it mainly occurs in men. miR-182-5p, a member of miR-183 family, acts as tumor suppressor or oncogene in various kinds of tumors. In this study, we first investigate that the absence of miR-182-5p in human bladder cancer promotes tumor growth by regulating the expression of Cofilin 1, an actin modulating-protein.

**Methods:**

Human bladder tumor tissue specimens were collected to detect the expression of miR-182-5p and Cofilin 1 by qRT-PCR. Luciferase activity assay was performed to demonstrate the regulation of Cofilin 1 mRNA 3′UTR by miR-182-5p. Then, cell experiments were performed to analysis the effect of miR-182-5p/Cofilin 1 pathway on tumor cell proliferation, migration, invasion and colony forming efficiency. Finally, xenograft tumor models were established to evaluate the role of miR-182-5p in tumorigenesis abilities in vivo.

**Results:**

qRT-PCR and Western blotting analysis showed that Cofilin 1 expression was up-regulated in both bladder cancer tissues and cell lines compared with normal. Luciferase activity assay showed that miR-182-5p specifically targets Cofilin 1 mRNA 3′UTR and represses the expression of Cofilin 1. Also, miR-182-5p inhibited bladder tumor cell proliferation, migration, invasion and colony forming efficiency. Furthermore, xenograft tumor model assay showed that miR-182-5p plays a negative role in bladder cancer tumorigenesis abilities in vivo.

**Conclusion:**

Present results suggest that miR-182-5p could inhibit human bladder tumor growth by repressing Cofilin 1 expression. Our findings may provide a new horizon for exploring therapeutic target of bladder cancer.

## Background

According to the global cancer statistics from 2018, bladder cancer caused estimated 549 393 new cases and 199,922 death in the year 2018. As the tenth most common cancer in the world, bladder cancer has an over 75% occurring in men. Smoking is the most determined risk factor for bladder cancer [1, 2].

MicroRNAs (miRNAs) are endogenous approximate 22 nt in length RNAs that can suppress target gene mRNA translation by sufficient or partial complementarity to 3′UTR of the mRNA [3]. miRNAs play crucial roles in multiple bio-progress, including cell proliferation, cell differentiation and cell death [4–6]. Furthermore, increasing studies showed that miRNAs have an intertwined pathway regulation in various human cancers by acting as tumor suppressors or oncogenes [7, 8]. miR-182-5p is a member of miR-183/96/182 cluster, and locates in the chromosome 7q31-34. In diverse kinds of tumors, miR-182-5p plays an implicated role through acting as a tumor suppressor or oncogene. Specifically, miR-182-5p act as tumor suppressor in renal cell cancer (RCC) [9], non-small cell lung cancer (NSCLC) [10, 11], osteosarcoma (OS) [12] and glioblastoma [13]. On the other hand, miR-182-5p is considered as oncogene in breast cancer [14], ovarian cancer [15] and prostate cancer [16].

Cofilin 1, a 19 kDa ubiquitous actin-modulating protein, is encoded by the non-muscle isoform of *CFL1* (Gene ID: 1072). It’s one of the three ADFs/Cofilin 1, including Cofilin 1, Cofilin 2 and ADF. Cofilin 1 is widely expressed in almost all mammal cell types, Cofilin 2 is mainly expressed in muscle tissues, and ADFs is expressed in brain and epithelial tissues [17]. Cofilin 1 acts as an important mediator of cell movement by controlling actin dynamics during cell protrusion [18, 19]. Since enhanced cell survival, metastasis and invasion extensively exist in tumor cell, activity of Cofilin 1, affected by expression level, phosphorylation level, pH and subcellular localization, closely correlates with tumorigenesis and tumor development [20, 21]. It has reported that an increasing expression of Cofilin 1 is observed in 70% prostate cancers, and expression of Cofilin 1 is suggested as an independent predictive factor [22]. Furthermore, Liu et al. [23] have showed that LMO2 enhances Cofilin 1 activity through inhibiting phosphorylation of Cofilin 1 by LIMK1, which ultimately promotes tumor cell invasion and metastasis in breast cancers. Therefore, Cofilin 1 may become a new potential tumor marker and target for treatment of malignant tumor [24–26].

In our earlier study, we found that Cofilin 1 expresses much higher in human bladder cancer tissues than para-tumor tissues, and suppressing Cofilin 1 by siRNA can inhibit tumor cell growth. Furthermore, we found that transcription factor 7-like 2 (TCF7L2) enhances Cofilin 1 expression by binding to Cofilin 1 promoter in human bladder cancer, which can promote tumor progress [25, 27]. Here, we expect to further explore the role of Cofilin 1 regulated by miR-182-5p in bladder cancer. Meanwhile we found that miR-182-5p can direct targets Cofilin 1 mRNA 3′UTR, and regulate the expression of Cofilin 1 in bladder cancer. The loss of miR-182-5p in bladder cancer induced a high level of Cofilin 1, which promoted tumor cell proliferation, migration and invasion and tumorigenesis abilities. The miR-182-5p/Cofilin 1 regulating axis reveals another potential mechanism of bladder cancer tumorigenesis.

## Materials and methods

### Tissue specimens

Eight pairs of bladder tumor and homologous para-tumor tissue samples were collected from the first people’s Hospital of Hainan. All samples were frozen and stored in liquid nitrogen until use. All patients signed a written consent, and this study approved by the institutional ethics committee of the first people’s Hospital of Hainan.

### RNA extraction and qRT-PCR analysis

Total RNA of tissues and cell lines were extracted using TRIzol reagent (Invitrogen, USA) according to the instructions. cDNA was synthesized using a ImProm-IITM Reverse Transcription System kit (Promega, USA). In detail, diluted 1 µg total RNA in 12 µl RNase free H_2_O, and incubated at 85 °C for 5 min, then rapidly cooled on ice for 5 min. In mRNA reverse transcription reaction, 0.5 µl Oligo (dT), 0.5 µl random primer, 2 µl 10 mM dNTP, 0.5 µl RNase inhibitor, 4 µl 5× buffer, 0.5 µl M-MLV reverse transcriptase were mixed with the RNA, then reacted at 30 °C for 10 min, 42 °C for 60 min, and 85 °C for 10 min. In mircoRNA reverse transcription reaction, 0.5 µl miR-182-5p-RT primer, 0.5 µl U6 primer, 2 µl 10 mM dNTP, 0.5 µl RNase inhibitor, 4 µl 5× buffer, 0.5 µl M-MLV reverse transcriptase were mixed with the RNA, then reacted at 42 °C for 60 min and 85 °C for 10 min. mRNA and miRNA expression level was quantified using a SYBR GREEN qPCR Super Mix kit (Invitrogen). 18srRNA was used for Cofilin 1 mRNA normalization and relative expression evaluation. U6 was used for miR-182 normalization and relative expression evaluation. The relative expression was evaluated using the 2^−ΔΔCt^ method. Each experiment was independently performed 3 times. The primers used are as follows: Cofilin 1-F: 5′-TTG TGC GGC TCC TAC TAA-3′, Cofilin 1-R: 5′-TTG CAT CAT AGA GGG CAT AG-3′, 18srRNA-F: 5′-CCT GGA TAC CGC AGC TAG GA-3′, 18srRNA-R: 5′-GCG GCG CAA TAC GAA TGC CCC-3′, miR-182-5p-RT: 5′-CTC AAC TGG TGT CGT GGA GTC GGC AAT TCA GTT GAG TGT GA-3′, miR-182-5p-F: 5′-ACA CTC CAG CTG GGT TTG GCA ATG GTA GAA CTC AC-3′, miR-182-5p-R: 5′-CTC AAC TGG TGT CGT GGA-3′, U6-F: 5′-CTC GCT TCG GCA GCA CA-3′, U6-R: 5′-AAC GCT TCA CGA ATT TGC GT-3′.

### Cell culture and transfection

The human embryonic kidney (HEK) 293T and human bladder cancer cell lines (RT4 and T24) were maintain in our lab. HEK293T, RT4 and T24 cells were cultured in DMEM (Hyclone, USA) supplied with 10% FBS (Hyclone) and penicillin/streptomycin (Hyclone). All cells were incubated at 37 °C in a humidified 5% CO_2_ incubator. 2 × 10^4^ cells were seeded in 24-well plates and cultured for 24 h, then, differentially transfected with plasmids or RNAs using Lipofectamine 2000 reagent (Invitrogen) according to the manufacturer’s instructions. Cofilin 1 was amplified from cDNA library and cloned into pCDNA 3.1 expression vector in our lab. The primers for Cofilin 1 amplification were as follows, forward: 5′-CCC AAG CTT GCC ACC ATG GCC TCC GGT GTG GCT GTC TCT G-3′, reverse: 5′-CCG GAA TTC TCA CAA AGG CTT GCC CTC CAG G-3′. Hsa-miR-182-5p mimics (Catalogue No. miR10000259-1-5) and has-miR-182-5p inhibitor (Catalogue No. miR20000259-1-5) were purchased from Ribo Bio Co., Ltd (Guangzhou, China).

### Western blotting

Prepared cells or tissues were added with pre-cooling RIPA (Beyotime Bio, Shanghai, China) lysate buffer applied with proteases inhibitor cocktail (Sigma, USA). Protein concentration were measured using BCA Protein Assay kit (Keygen Biotech, Nanjing, China). Total proteins were mixed with 5× SDS-PAGE loading buffer and heat to 100 °C for 10 min, then, were separated with 4–15% SDS-PAGE and transferred to PVDF membranes (Millipore, USA). After blocked with 5% non-fat milk for 1 h, the PVDF membranes were incubated with specific primary and secondary (horseradish peroxidase, HRP-conjugated) antibodies. Finally, the protein bands were visualized using chemiluminescence HRP substrate (Millipore) in a dark room. The primary antibodies used are anti-GAPDH (1:10000, Kangchen, Shanghai, China) and anti-Cofilin 1 (1:1000, Abcam, USA). The secondary antibody used is HRP-conjugated goat anti-rabbit IgG (1:20000, Southern biotech, China).

### Luciferase reporter assay

Wild type (wt) 3′UTR fragment of Cofilin 1 containing miR-182-5p binding site was amplified and cloned into psi-CHECK-2 luciferase reporter vector (Promega). A mutant type (mut) of 3′UTR fragment of Cofilin 1, mutation within the miR-182-5p binding site, also was cloned into psi-CHECK-2 vector as control. Reporter-vectors, Cofilin 1 ectopic expression vectors and RNAs were respectively transfected into HEK 293T cells. 48 h after transfection, cells were lysed with passive lysis buffer. Then, luciferase activity was measured by GloMax 20/20 (Promega) detector using the Dual-Luciferase Reporter Assay System (Promega) according to the instructions.

### Cell viability assay

Cell viability was measured using cell count kit-8 (CCK8, Beyotime, China). 2 × 10^3^ cells were seeded into 96-well plates, and incubated for 24 h. After proper plasmids or RNAs transfection, cell viability was measured every day in the following 4 days. 10 µl CCK8 solutions was supplied into each of the 96-well plates. After 1.5 h 37 °C 5% CO_2_ incubation, cells were subjected to measure absorbance at 450 nm using an automatic absorbance microplate reader (Bioteke, Beijing, China). Each experiment was independently performed 3 times.

### Cell cycle assay

Cells were collected at least 1 × 10^6^ in number, washed with pre-cooling PBS twice, fixed with 70% ethanol at 4 °C overnight. Then, cells were washed with pre-cooling PBS, incubated with 0.5 ml PBS, 50 µg/ml PI, 0.5% RNase A and 0.02% Triton X-100 at 4 °C without light for 10 min. Finally, cell cycle was analyzed by flow cytometry (BD Biosciences, SanJose, USA).

### Cell migration and invasion assays

Tumor cell migration and invasion were determined using an 8 µm pore size membrane in trans-well chamber (BD Biosciences). In cell migration assay, 1 × 10^5^ cells were seeded in upper chamber, 0.6 ml medium containing 10% FBS was applied to the lower chamber as a chemoattractant. After 24 h 37 °C 5% CO_2_ incubation, the cells on the upper chamber were removed with a cotton swab. Migration cells were fixed with 4% paraformaldehyde and stained with crystal violet. Cell images were captured using an Olympus (Japan) microscope at 100× magnification, and analyzed using ImageJ 1.44 software (Java). In cell invasion assay, the trans-well membrane was coated with matrigel (BD Biosciences). The follow procedures were same as cell migration assay. Each experiment was independently performed 3 times.

### Cell colony formation assay

Transfected cells were digested and counted. 300 cells were seeded in each well of 6-well plates containing 2 ml 10% FBS medium. After 7 days 37 °C 5% CO_2_ incubation, colonies were fixed with 4% paraformaldehyde and stained with crystal violet. Then, colony images were captured, and the number of colony was counted. The ratio of colony counts and inoculated cell number regarded as the colony-forming efficiency.

### Xenograft tumor model assay

Four to five week-old BCLB/C nude mice were obtained from Vital River Laboratory Animal Technology Co., Ltd (Beijing, China). Mice were randomly divided into 4 groups (n = 6). miR-182-5p mimics, miR-182-5p inhibitor and Cofilin 1 expression plasmids were respectively transfected into RT4 cells for 48 h, and 1 × 10^7^ cells were respectively collected and subcutaneously injected into mouse left dorsal flank. Then, tumor width (W) and length (L) were measured every few days. Tumor volume was represented as L × W^2^/2. On day 44, mice were executed by cervical dislocation, and the tumors were excised to weigh.

### Statistical analysis

Data in this study were analyzed and exhibited using Origin 8.5 (OriginLab, USA) software and shown as mean ± SD. Difference level between groups were evaluated by Student’s t-test. *p *< 0.05 was regarded as statistically significant.

## Results

### Cofilin 1 is up-regulated in human bladder cancer tissues and cell lines

**Fig. 1:**
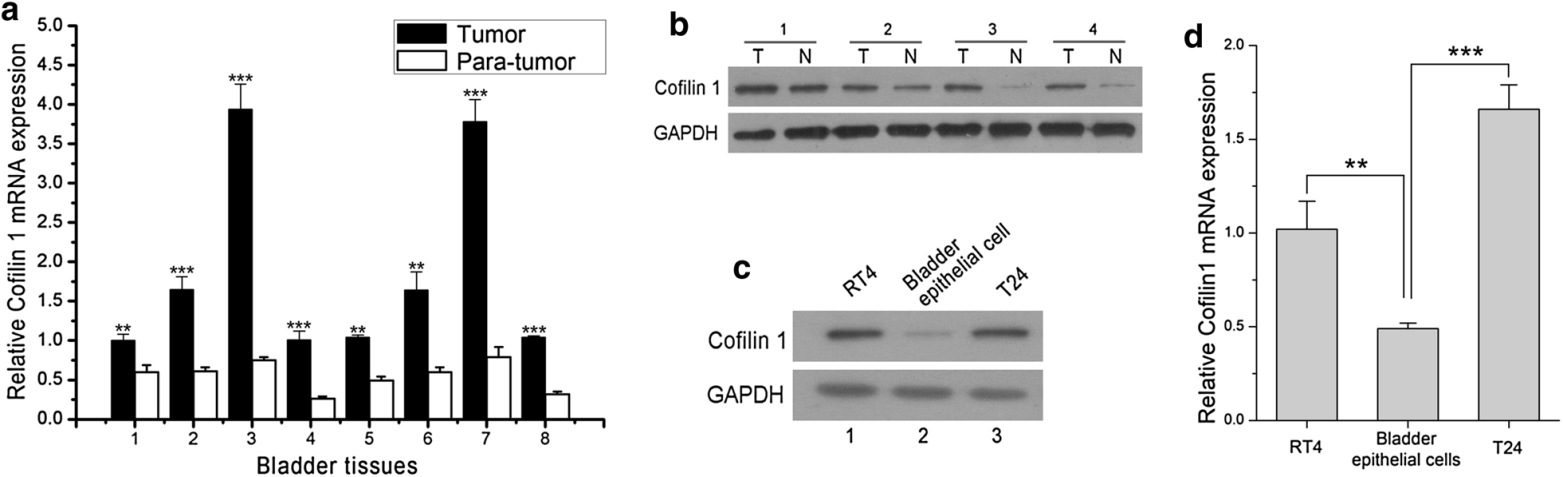
Cofilin 1 upregulates in bladder tumor tissues and cell lines. **a** Cofilin 1 mRNA expression in bladder tumor and homologous para-tumor tissues were measured by qRT-PCR. **b** Cofilin 1 protein expression in bladder tumor (T) and homologous para-tumor (N) tissues were measured by Western blotting. **c**, **d** Cofilin 1 mRNA and protein expression in bladder cancer cell lines (RT4 and T24) and normal bladder epithelial cell line (SV-Huc-1) measured by qRT-PCR and Western blotting. ** Indicates *p *< 0.01, ***indicates *p *< 0.001

We firstly measured the expression of Cofilin 1 in human bladder cancer tissues and cell lines. In eight human bladder cancer tissue samples, Cofilin 1 mRNA expression remarkably increased compared with homologous para-cancer tissues (Fig. 1a). Similarly, Western blotting analysis revealed a higher Cofilin 1 protein level in bladder cancer tissues than in adjacent normal tissues (Fig. 1b). Furthermore, we determined Cofilin 1 expression in two human bladder cancer cell lines, RT4 and T24. Compared with bladder epithelial cells, Cofilin 1 expressed much higher in RT4 and T24 cells both in mRNA and protein level (Fig. 1c, d). These data suggest that the high expression of Cofilin 1 in human bladder cancer correlates to bladder tumorigenesis.

### miR-182-5p direct targets Cofilin 1 mRNA 3′ UTR and regulates gene expression

**Fig. 2:**
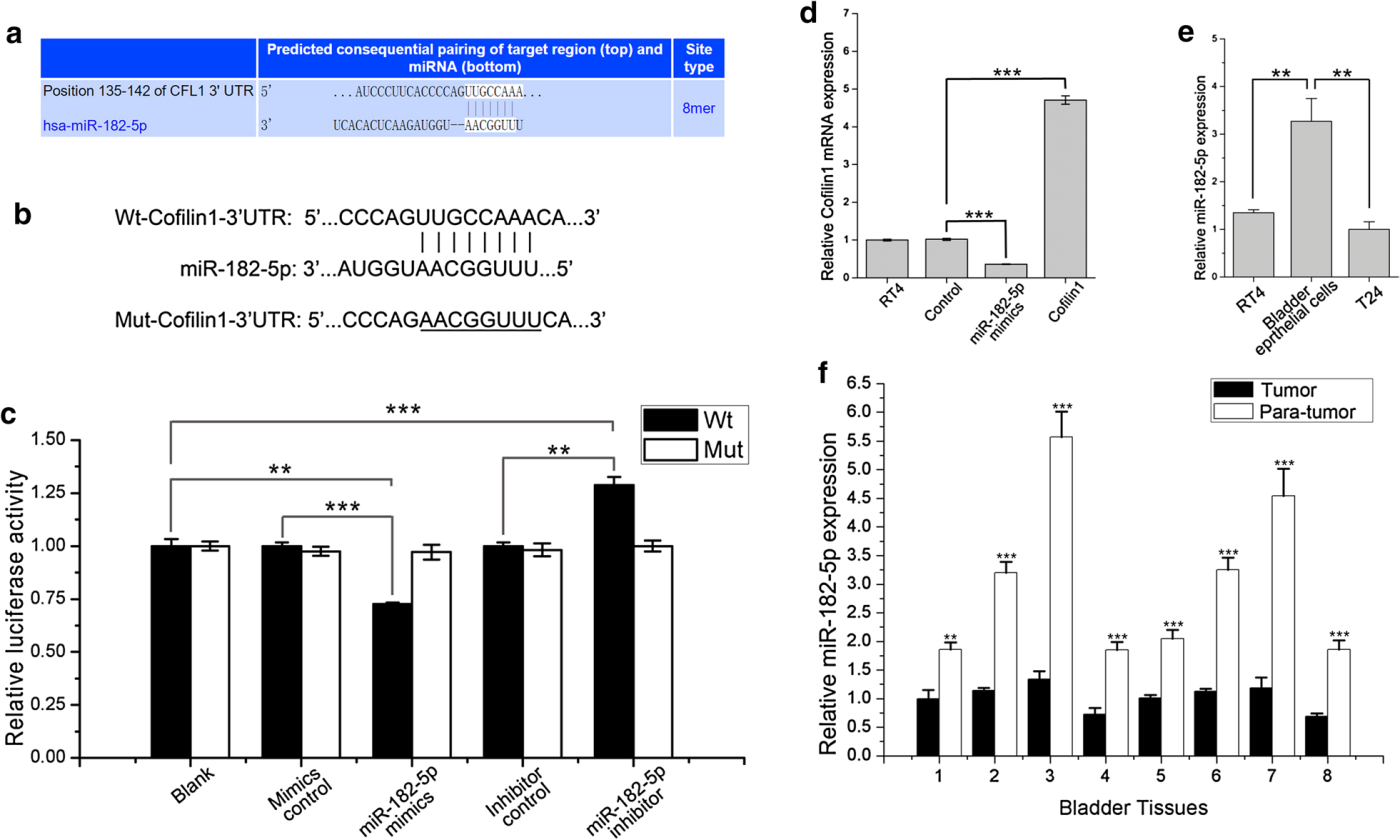
miR-182-5p direct targets Cofilin 1 mRNA 3′UTR. **a** TargetscanHuman 7.2 analysis showed that Cofilin 1 mRNA 3′UTR contains a predicted miR-182-5p binding site at position 135-142. **b** Wt-Cofilin1-3′UTR sequence complementarity to miR-182-5p at position 135-142, and Mut-Cofilin1-3′UTR has a mutant at miR-182-5p binding site. **c** Relative luciferase activity was measured after miR-182 mimics, miR-inhibitor, wt-Cofilin 1-3′UTR-psi-CHECK-2 and mut-Cofilin 1-3′UTR-psi-CHECK-2 plasmids transfection. **d** Cofilin 1 mRNA expression was measured after miR-182 mimics and Cofilin 1 expression plasmids transfection by qRT-PCR. **e** miR-182 mRNA level was measured in bladder tumor and homologous para-tumor tissues by qRT-PCR. **f** miR-182 expression level was measured in bladder cancer cell lines (RT4 and T24) and normal bladder epithelial cell line (SV-Huc-1) measured by qRT-PCR. ** Indicates *p *< 0.01, ***indicates *p *< 0.001

miR-182-5p plays an important role in various kinds of tumors by participating in multiple cell signal pathways [10, 12, 28]. First, professional share databases (TargetscanHuman and PicTar) were used to predict target genes of miR-182-5p. *CFL1* was one of miR-182-5p predicted target genes, and had a relative good efficiency score of prediction (Fig. 2a). To further confirm miR-182-5p targeting gene *CFL1*, wild and mutant Cofilin 1 mRNA 3′TUR were respectively constructed into psi-CHECK2 vector (Fig. 2b), then luciferase activity assay was performed. In wt-Cofilin 1-3′UTR group, luciferase activity was markedly repressed by miR-182-5p mimics transfection, and promoted by miR-182-5p inhibitor transfection. However, the luciferase activity have no significant difference compared with control group when either promoting or interference miR-182-5p expression in mut-Cofilin 1–3′UTR groups (*p *> 0.05) (Fig. 2c). In RT4 cells, Cofilin 1 mRNA expression was down-regulated with miR-182-5p mimics transfection, and up-regulated with miR-182-5p inhibitor transfection (Fig. 2d). All these results indicate that miR-182-5p can negatively regulate Cofilin 1 expression by binding to gene 3′UTR at position 135–142.

### miR-182-5p is down-regulated in human bladder cancer tissues and cell lines

miR-182-5p level was measured in 8 human bladder cancer tissue samples by qRT-PCR. The results showed that expression of miR-182-5p was decreased in bladder cancer tissues compared with homologous para-tumor tissues (Fig. 2f). Similarly, miR-182-5p expression reduced in both RT4 and T24 cells compared with normal bladder epithelial cells (Fig. 2e). These results indicate that low level of miR-182-5p contributes to an up-regulation expression of Cofilin 1 in bladder cancer cells.

### miR-182-5p reduces bladder cancer cell proliferation through promoting Cofilin 1

**Fig. 3:**
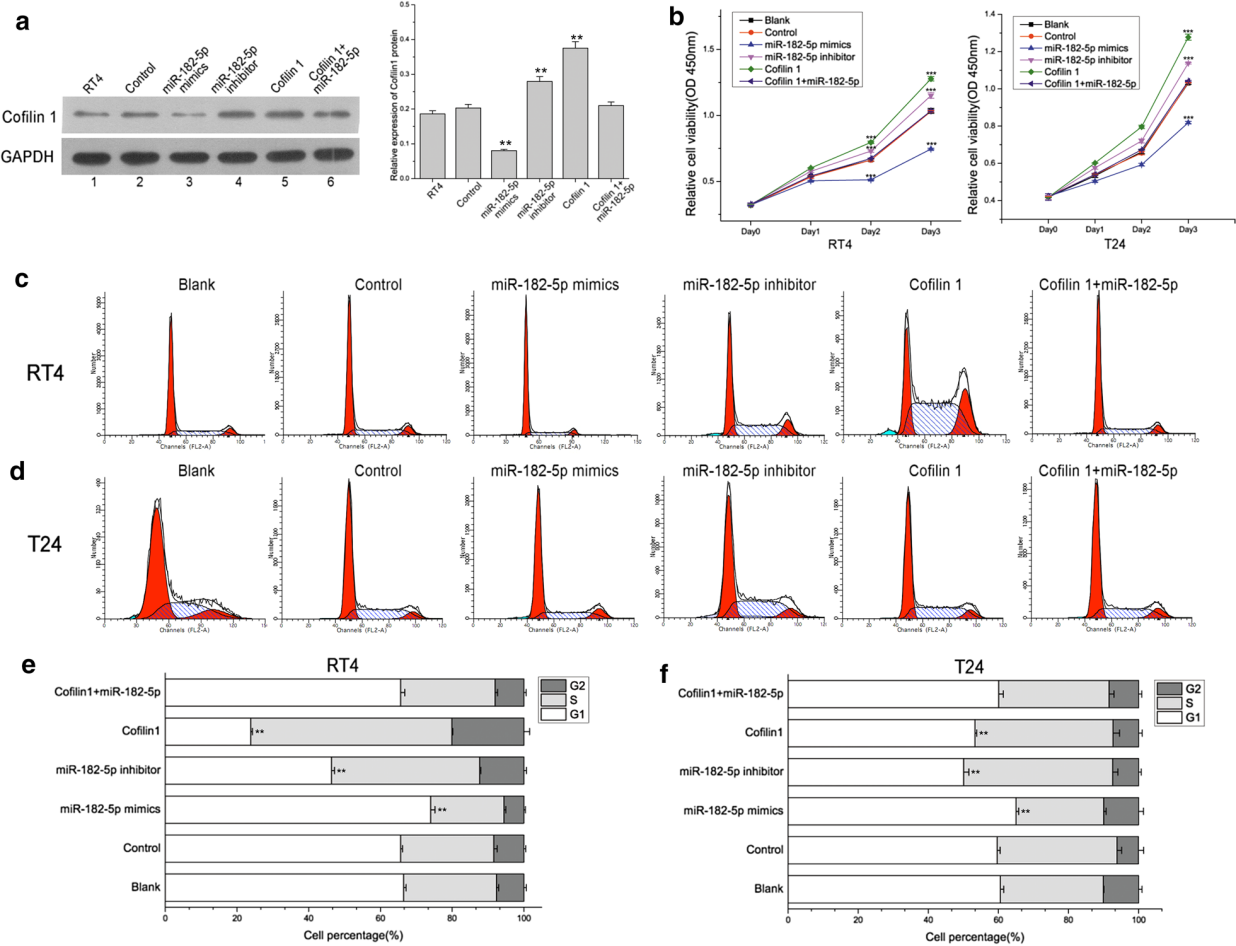
miR-182 inhibits bladder cancer cell growth through repressing Cofilin 1. **a** Cofilin 1 expression was measured by Western blotting after miR-182 mimics, miR-182inhibitor and Cofilin 1 vectors transfection in RT4 cells. **b** Cell proliferation were determined by CCK-8 assay after miR-182 mimics, miR-182 inhibitor and Cofilin 1 plasmids transfection in RT4 and T24 cells. **c**, **d** Cell cycle was determined after miR-182 mimics, miR-182 inhibitor and Cofilin 1 vectors transfection in RT4 and T24 cells. **e**, **f** Data analysis of cell cycle in RT4 and T24 cells. * Indicates *p *< 0.05, ** indicates *p *< 0.01, ***indicates *p *< 0.001

Since miR-182-5p could direct targets Cofilin 1 mRNA 3′UTR, we next observed the Cofilin 1 level after miR-182-5p mimics, miR-182-5p inhibitor, Cofilin 1 and miR-182-5p mimics + Cofilin 1 transfection in RT4 cells by Western blotting. The results showed that miR-182-5p plays a negative role in regulating Cofilin 1, and the overexpression of Cofilin 1 could be partly reverted by miR-182-5p co-transfection (Fig. 3a). In CCK8 assay, cell proliferation was inhibited by miR-182-5p mimics transfection, and promoted by miR-182-5p inhibitor transfection. Cofilin 1 transfection could remarkably promote RT4 and T24 cell proliferation, however, this promotion effect was counteracted by miR-182-5p co-expression (*p *< 0.0001) (Fig. 3b). In cell cycle assay, miR-182-5p expression blocked cells at G1 phase of cell cycle, in contrast, miR-182-5p inhibitor transfection increased the cell percentage of S and G2 phase. Cofilin 1 expression could significantly promote cells to transfer from G1 to S and G2 phase. Also, this promotion effect was counteracted by miR-182-5p co-expression (Fig. 3c–f). These data suggest that the loss of miR-182-5p in bladder cancer cell can promote cell proliferation viability by accelerating Cofilin 1 expression.

### miR-182-5p represses bladder cancer cell migration, invasion and colony formation ability through promoting Cofilin 1

**Fig. 4:**
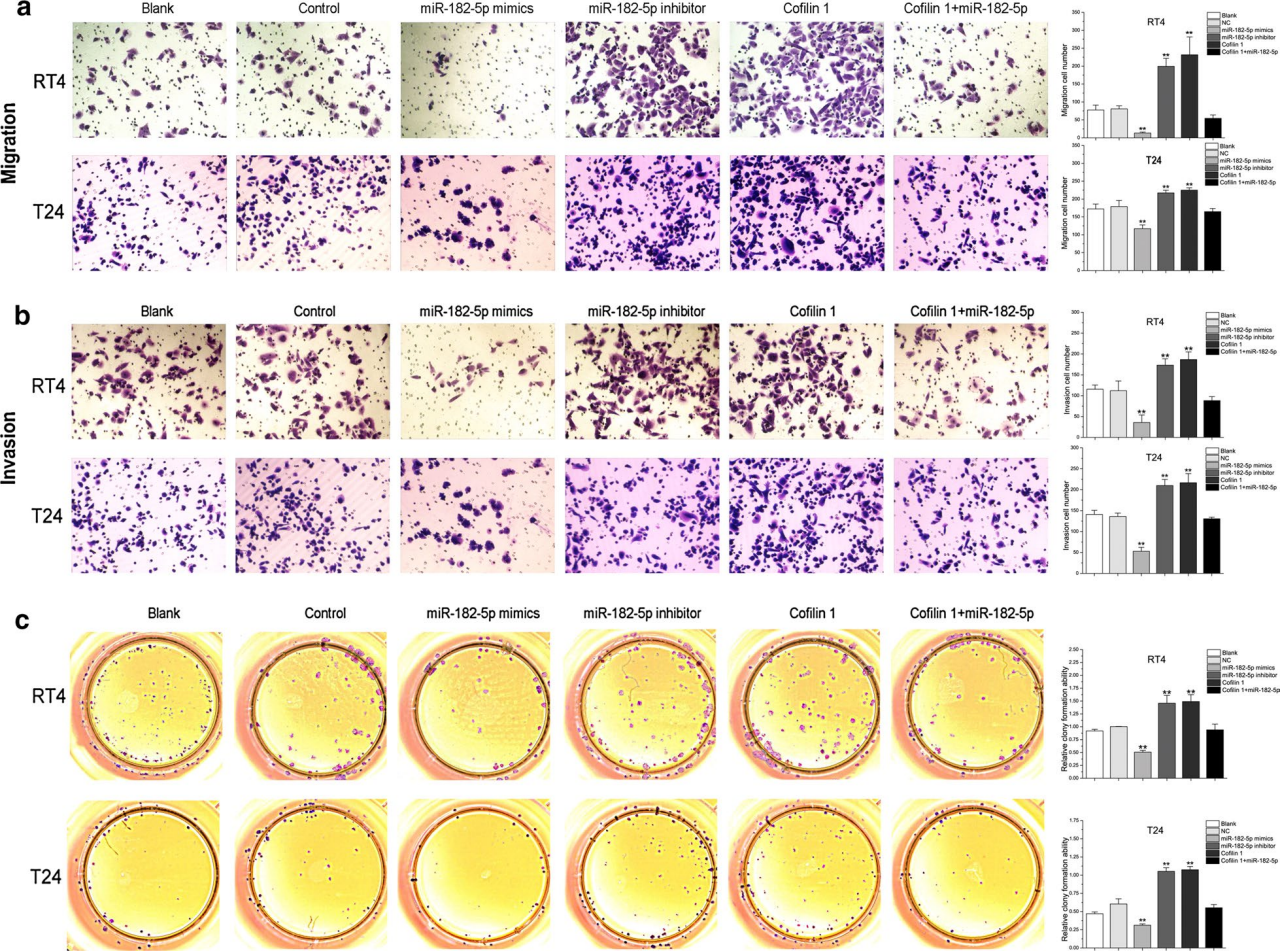
miR-182 inhibits bladder cancer cell migration, invasion and colony forming efficiency through repressing Cofilin 1. **a**, **b** Cell migration and invasion were measured by using trans-well membrane and crystal violet staining after transfection of miR-182 mimics, miR-182 inhibitor and Cofilin 1 expression vectors in RT4 and T24 cells. **c** Cell colony forming efficiency was measured after transfection of miR-182 mimics, miR-182 inhibitor and Cofilin 1 expression vectors in RT4 and T24 cells. ** Indicates *p *< 0.01

Next, the influence of miR-182-5p and Cofilin 1 on RT4 and T24 cell migration and invasion were evaluated using trans-well membrane. Compared with control and blank (RT4 and T24) groups, miR-182-5p mimics transfection suppressed cell migration and invasion, Cofilin 1 expression and miR-182-5p inhibition enhanced the ability of cell migration and invasion. While miR-182-5p and Cofilin 1 was co-expressed, the number of migration and invasion cells had no significant difference compared with control and blank groups (Fig. 4a, b). In colony formation assay, miR-182-5p inhibition and Cofilin 1 expression had a higher colony forming efficiency than control and blank groups. miR-182-5p mimics and Cofilin 1 co-transfection converted the colony forming ability to normal level (*p *> 0.01) (Fig. 4c). Thus, the low level of miR-182-5p in bladder cancer also facilitate cell migration, invasion and colony forming efficiency through promoting Cofilin 1.

### Effects of miR-182-5p on xenograft tumor growth in nude mice

**Fig. 5:**
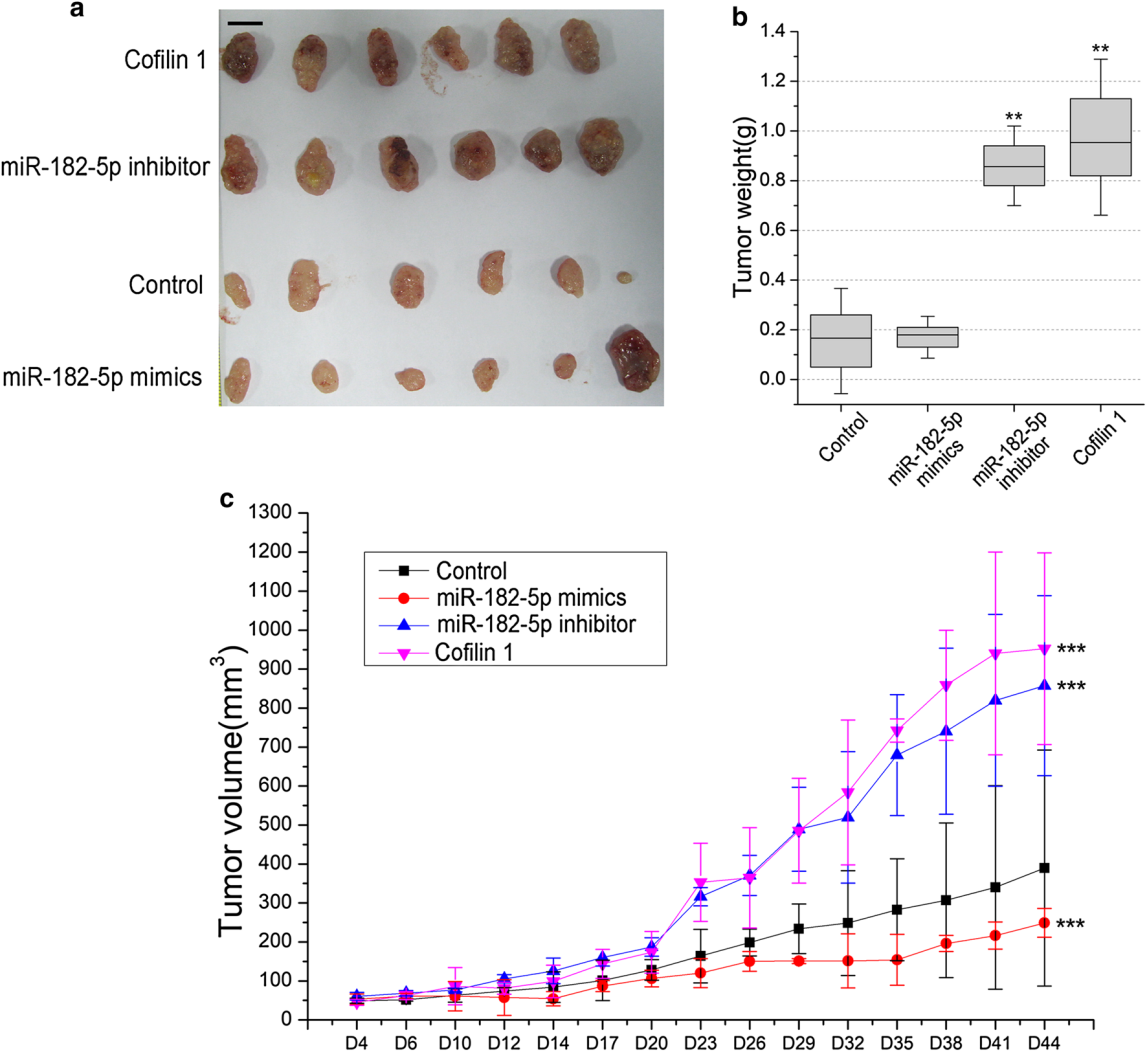
The effect of miR-182 on RT4 xenograft tumor growth, volume and weight. **a** On day 44, the RT4 xenograft mice were executed by cervical dislocation, and tumors were excised to weigh. Scale bar, 1 cm. **b** Tumor weight was measured on day 44. **c** Tumor growth and volume in RT4 xenograft model were determined every few days. ** Indicates *p *< 0.01

For further investigating the functions of miR-182-5p in bladder cancer, xenograft tumor assay was performed. miR-182-5p mimics, miR-182-5p inhibitor and Cofilin 1 expression vectors transfected RT4 cells were injected into nude mice, then, tumor volume was determined every few days. The results showed that xenograft tumor volume and growth of miR-182-5p inhibitor and Cofilin 1 groups were obviously increased compared with control (RT4, *p *< 0.05) (Fig. 5c). On day 44, xenograft tumors were excised to weigh (Fig. 5a). Also, xenograft tumor of miR-182-5p inhibitor and Cofilin 1 groups are more weight than control (*p *< 0.01) (Fig. 5b). No significant difference of xenograft tumor weight was observed between miR-182-5p mimics and control groups (*p *> 0.05). But, paired-samples *t*-test statistical analysis in samples that tumor volume on day 4 to day 44 showed that there is a significant statistical difference (*p *= 0.0009) between miR-182 mimics and control groups. So we considered that miR-182 mimics transfection can suppresses tumor growth in xenografts (Fig. 5c).

## Discussion

With the increasing and aging of population, cancer has growing incidence and mortality worldwide, and has become a heavy burden on society especially in developing countries. Human bladder cancer is one of the most common cancer with an incident of 549,393 in the year 2018 [2]. Not smoking, schistosomiasis controlling, more fruit, vegetables taking in and proper treatment are the major prevention measures for bladder cancer [29].

Cofilin 1, belongs to the actin depolymerizing factor (ADF), is a ubiquitous protein that binds to actin, participates in directed cell movement in response to chemoattractant or stimulation by involving in actin dynamics at plasma membrane during cell protrusion [18, 30, 31]. Also, Kanellos et al. [32] indicated that Cofilin 1 involves in maintaining tissue homeostasis and promoting cell survival by preventing the cell nucleus from being damaged by actin contractility. In our study, we found that expression of Cofilin 1 accelerates tumor cell proliferation and decreases cells of G1-arrested. So we consider that Cofilin 1 accelerate cell proliferation mainly through enhancing stress tolerance of cells and promoting movement into the beneficial environment. In cancer cells, Cofilin 1 activity, which is affected by factors of phosphorylation level, pH, subcellular localization and binding of phosphoinositides, is necessary for tumor cell motility and invasion. Notably, LIM kinase 1 (LMK1), which can phosphorylates and inactivates Cofilin 1, and Cofilin 1 were simultaneously increased in invasive cells [33]. Interfering the expression of Cofilin 1 in cancer cell inhibit cell invasion by weaken the maturation and stability of invadopodia [34]. Wang et al. [35] reported that Cofilin 1 overexpressed in invasive subpopulation of cancer cells from the primary tumor. In bladder cancer, Patrick et al. [36] proved that with the increasing of tumor grade, Cofilin 1 expression was significant elevated, and localization of Cofilin 1 to nucleus also increased. However, the mechanism of Cofilin 1 up-regulated in cancer cells was not presented in these studies. In our earlier study, we reported that transcription factor TCF7L2 can binds to Cofilin 1 promoter and increases the gene expression in bladder cancer, which promotes the tumor progress [25, 27]. Here, we found another regulated pathway by miR-182-5p that can elevate levels of Cofilin 1 in tumor cells.

It is well known that miRNAs have close correlation with tumorigenesis through participating in multiple bio-process, including cell proliferation, migration, invasion and apoptosis [7, 37, 38]. As a member of miR-183 family, miR-182-5p plays a key and complex role in diverse kinds of tumors by acting as oncogene and tumor suppressor. For instance, miR-182-5p inhibits renal cell cancer (RCC) cell proliferation, invasion and apoptosis by regulating PI3K/AKT/mTOR pathway [9]; miR-182-5p suppresses non-small cell lung cancer (NSCLC) cell proliferation, invasion and invadopodia formation by targeting cortical, an actin-associated protein [10, 39]; miR-182 reduces proliferation of human osteosarcoma cell (OS) by targeting HOXA9 [12]; miR-182 acts as an oncogene and promote hepatocellular cancer (HCC) progression by targeting FOXO3a [40]; In gastric adenocarcinoma, miR-182 inhibit tumor growth by targeting cAMP responsive element binding protein 1 (CREB1) [41]. Due to the numerous miR-182 target genes, the mechanisms of miR-182 involving in tumorigenesis is quite complex, even be inconsistent. At present, the research about miR-182-5p functions in human bladder cancer is rare, and the mechanism involved in is unclear.

In this study, we demonstrated that Cofilin 1 is overexpressed in human bladder cancer tissues and cell lines compared with normal tissues or epithelial cells, which is consistent with our earlier research [25, 27]. miR-214-5p direct targets Cofilin 1 mRNA 3′UTR and regulates the gene expression. Therefore, decline expression of miR-182-5p contributes to the high level of Cofilin 1 in bladder cancer, for which enhanced the tumor cell proliferation, migration and invasion and tumorigenesis abilities. In xenograft mice model assay, miR-182-5p inhibition observably increased the xenograft tumor growth compared with control. Also, miR-182-5p mimics transfection is able to suppress the xenograft tumor growth.

## Conclusions

We first proved that Cofilin 1 is a direct target of miR-182-5p in human bladder cancer. Following with the demonstrating of TCF7L2/Cofilin 1 regulating pathway in bladder cancer, we uncovered another regulating mechanism of which Cofilin 1 promoting tumor progress through miR-182-5p/Cofilin 1 regulating axis. Loss of miR-182-5p in bladder cancer can promotes Cofilin 1 expression, which may have a potential diagnostic and targeted therapy value for bladder cancer.

## Abbreviations

miRNAs: microRNAs
CFL1: Cofilin 1
TCF7L2: transcription factor 7-like 2
ADF: actin depolymerizing factor
